# Randomised controlled trial of the Community Navigator programme to reduce loneliness and depression for adults with treatment-resistant depression in secondary community mental health services: trial protocol

**DOI:** 10.1186/s13063-023-07684-4

**Published:** 2023-10-06

**Authors:** Theodora Stefanidou, Gareth Ambler, Gergely Bartl, Nick Barber, Jo Billings, Tumelo Bogatsu, Richard Carroll, Beverley Chipp, Maev Conneely, Anne-Marie Downey, Gamze Evlat, Rachael Hunter, Marie Le Novere, Glyn Lewis, Tanya Mackay, Steven Marwaha, Zubair Matin, Georgia Naughton, Chandani Nekitsing, Millie O’Sullivan, Vanessa Pinfold, Shengning Pan, Angela Sobers, Keith J. Thompson, Jerusaa Vasikaran, Martin Webber, Sonia Johnson, Brynmor Lloyd-Evans

**Affiliations:** 1 Division of Psychiatry, University College London, Maple House, 149 Tottenham Court Road, London, W1T 7NF UK; 2 Department of Statistical Sciences, University College London, London, UK; 3 Priment Clinical Trials Unit, University College London, London, UK; 4 The McPin Foundation, London, UK; 5 Institute for Mental Health, University of Birmingham, Birmingham, UK; 6 Tees, Esk and Wear Valley, NHS Foundation Trust, Durham, UK; 7 Camden and Islington NHS Foundation Trust, London, UK; 8 Department of Primary Care and Population Health, University College London, London, UK; 9 School for Business and Society, University of York, York, UK; 10 Barnet, Enfield and Haringey, NHS Mental Health Trust, London, UK

**Keywords:** Loneliness, Depression, Community navigator, Randomised controlled trial

## Abstract

**Background:**

New treatments are needed for people with treatment-resistant depression (TRD), who do not benefit from anti-depressants and many of whom do not recover fully with psychological treatments. The Community Navigator programme was co-produced with service users and practitioners. It is a novel social intervention which aims to reduce loneliness and thus improve health outcomes for people with TRD. Participants receive up to 10 individual meetings with a Community Navigator, who helps them to map their social world and set and enact goals to enhance their social connections and reduce loneliness. Participants may also access group meet-ups with others in the programme every 2 months, and may be offered modest financial support to enable activities to support social connections.

**Methods:**

A researcher-blind, multi-site, 1:1 randomised controlled trial with *N* = 306 participants will test the effectiveness of the Community Navigator programme for people with TRD in secondary community mental health teams (CMHTs). Our primary hypothesis is that people who are offered the Community Navigator programme as an addition to usual CMHT care will be less depressed, assessed using the PHQ-9 self-report measure, at 8-month, end-of-treatment follow-up, compared to a control group receiving usual CMHT care and a booklet with information about local social groups and activities. We will follow participants up at end-of-treatment and at 14 months, 6 months after end-of-treatment follow-up. Secondary outcomes include the following: loneliness, anxiety, personal recovery, self-efficacy, social network, social identities. We will collect data about health-related quality of life and service use to investigate the cost-effectiveness of the Community Navigator programme.

**Discussion:**

This trial will provide definitive evidence about the effectiveness and cost-effectiveness of the Community Navigator programme and whether it can be recommended for use in practice. The trial is due to finish in August 2025.

**Trial registration:**

Prospectively registered on 8th July 2022 at: ISRCTN13205972.

**Supplementary Information:**

The online version contains supplementary material available at 10.1186/s13063-023-07684-4.

## Administrative information

Note: the numbers in curly brackets in this protocol refer to SPIRIT checklist item numbers. The order of the items has been modified to group similar items (see http://www.equator-network.org/reporting-guidelines/spirit-2013-statement-defining-standard-protocol-items-for-clinical-trials/).

**Table:** 

Title {1}	Randomised controlled trial of the Community Navigator programme to reduce loneliness and depression for adults with treatment resistant depression in secondary mental health services
Trial registration {2a and 2b}.	ISRCTN13205972 https://doi.org/10.1186/ISRCTN13205972
Protocol version {3}	7th July 2023, Version 4.0
Funding {4}	The trial is funded through the English National Institute for Health Research, Health Technology Assessment (HTA) Programme
Author details {5a}	See author list above
Name and contact information for the trial sponsor {5b}	Camden and Islington NHS Foundation Trust: sponsor.noclor@nhs.net
Role of sponsor {5c}	The study sponsors review study design; the collection, management, analysis, and interpretation of data; writing of the report; and the decision to submit the report for publication. Decisions about study design and conduct are made by the study researchers, but the sponsors’ approval is required.

## Introduction

### Background and rationale {6a}

About a third of people with depression who take anti-depressants with appropriate treatment protocols do not experience improvement and their depression can be termed “treatment resistant” [1, 2]. Psychological treatments are effective for some but of limited benefit for many in this treatment-resistant depression (TRD) group: symptom remittance was achieved for less than half the participants in a large trial of cognitive behavioural therapy for TRD [3]. Among people with TRD in observational studies, only 20–30% recover over a few years [4]. For people with TRD whose illness is already protracted, only about 40% recover over 10 years’ follow-up [5]. Many of those with TRD who have the most severe, complex, and enduring difficulties are supported in the UK by secondary mental health services (where people may be referred from GP, other primary care or mental health crisis services). In a UK trial, optimised medical and psychological support for TRD from specialist teams provided only modest additional benefit compared to routine secondary care [6]. A completed feasibility trial which informs this trial protocol confirmed the high levels of clinical and social need in our trial target population of people with TRD in secondary care services: participants were typically single, not in work, met clinical thresholds for severe depression and anxiety, and were extremely lonely [7]. More research and new types of support are urgently needed for this clinical group.

The social determinants of depression [8, 9], including the important role of social relationships [10, 11], have long been understood, yet we lack established social interventions of known effectiveness. Loneliness, defined as the subjectively experienced gap between desired and actual social relationships [12], is an independent predictor of recovery from depression [13]. People with depression have tenfold increased odds of loneliness compared to the general population [14] and are commonly extremely lonely [15]. Yet support with social relationships and loneliness is often overlooked in mental health services [16] and we lack well-developed, effective programmes [17, 18]. Reducing loneliness offers a promising intervention target, different from those addressed by pharmacological or psychological treatments for depression, for improving outcomes for people with TRD.

The Community Navigator programme is a novel social intervention designed to reduce depression severity by reducing loneliness and social isolation for people with TRD in secondary care. A completed feasibility trial of the Community Navigator programme indicated that the programme was feasible to deliver and evaluate, and was well-received by participants [7, 19]. The direction and magnitude of effect suggested for loneliness and depression outcomes showed promise [7] and warranted a definitive trial to test the effectiveness of the Community Navigator programme. The trial described in this protocol will test the programme’s effectiveness and cost-effectiveness.

### Objectives {7}

The primary objective is to test the hypothesis that people with TRD in secondary care community mental health services who are randomised to be offered the Community Navigator programme in addition to routine care, will be less depressed, measured using the Patient Health Questionnaire – Mental Health Disorders (PHQ-9) scale [20], at 8-month (end-of-treatment) follow-up, compared to a control group receiving routine care and an information booklet about local social resources.

Secondary objectives include testing the hypotheses that, compared to controls, the intervention group will be less depressed at 14 months (6 months after end-of-treatment) follow-up, and less lonely, less anxious, with better personal recovery at 8- and 14-month time-points. A further objective is to determine the cost-effectiveness of the Community Navigator programme.

In an embedded sub-study, we will explore, through qualitative interviews, the perceived impact of the Community Navigator programme, how benefits were achieved, and key considerations for its provision in NHS settings. Methods and findings from the qualitative sub-study will be reported in a separate publication, outside of this trial protocol.

### Trial design {8}

This is a researcher-blind, parallel group randomised controlled superiority trial with two arms. Eligible participants will be randomised in a 1:1 allocation ratio to receive either support from a Community Navigator in addition to routine care (treatment arm), or a booklet with information about local groups and resources plus routine care (control group).

Assessments will be conducted by blinded researchers at the time of consent (baseline) and at two main outcome points: 8-month (end-of-treatment) and 14-month (6 months after end-of-treatment). Baseline measures will be collected at the screening visit, or may be completed if necessary in subsequent meetings, within a maximum of 4 weeks from taking consent. Additional depression and loneliness ratings will be collected through a self-completed online form or through a phone or video call with a study researcher at 4 and 11 months. These additional 4- and 11-month data collection points will be used in additional analyses exploring mediating effects of loneliness on depression (see section “Methods for additional analyses (e.g., subgroup analyses) {20b}”).

## Methods: participants, interventions and outcomes

### Study setting {9}

The trial is being conducted in England and patients are recruited through Community Mental Health Teams (CMHTs) or equivalent secondary mental health care services. Depending on local service configurations, participating services may be general CMHTs for adults with any mental health diagnosis, or teams specifically for patients with depression or anxiety. General adult CMHTs or older adult CMHTs may both be included, as long as they serve patients meeting the trial inclusion criteria.

### Eligibility criteria {10}

We will recruit adults with TRD using a participating mental health team.

Inclusion criteria:

1. Age 18 + 
2. Meet ICD-10 diagnostic criteria for depression assessed using the Clinical Interview Schedule-Revised interview (CIS-R)
3. Have had at least two reported courses of anti-depressants without symptom remission, confirmed by the participant
4. Score of 2 or more on 6-item De Jong Gierveld Loneliness Scale (DJG-6) loneliness scale (a minimum threshold score for being lonely)

Exclusion criteria:

1. Are due to be discharged from the mental health team within the trial intervention period (8 months)
2. Currently using mental health inpatient services
3. Identified by involved clinicians or clinical records as having a primary diagnosis of a serious mental illness other than TRD, defined as schizophrenia or other non-mood psychotic disorders (ICD-10 codes F20-29 or equivalent) or bipolar disorder (ICD-10 code F31 or equivalent), or a diagnosis of dementia (ICD-10 codes F00-F03 or equivalent) or mild cognitive impairment (ICD-10 code F06.7 or equivalent)
4. Lacks capacity to consent to participate
5. Does not understand English well enough to give informed consent and engage with the study intervention
6. Has a care coordinator (the main clinician providing case management) who is the supervisor of the Community Navigators working within the CMHT team.

Exclusion criteria will be checked with the referring NHS staff member and with the participant during eligibility screening with the study researcher. Dementia and mild cognitive impairment diagnoses were explicitly added as exclusion criteria in an amendment to the original approved protocol, when recruitment was planned in older adults CMHTs in one participating site. Please see Additional file 1: Appendix 1 for a summary of protocol amendments.

### Who will record informed consent {26a}

Study researchers will ask clinicians or local research staff in participating teams to identify potential eligible participants and ask them about willingness to talk to a researcher. Clinicians will review their caseloads to identify potential eligible participants. Local research staff will screen case registers and clinician caseloads in adherence with local Trust privacy notice permissions and consent to contact arrangements. A study researcher, trained in Good Clinical Practice guidelines [21], will contact potential participants who have been referred, provide more information about the study and a written information sheet and answer questions, then seek informed consent.

Consent will be sought at least 24 h after the person has been given the study documentation, via a written, signed consent form or verbally via a recorded video call or phone call. Following consent, the study researchers will conduct the final stage of eligibility screening (described in the “Outcomes {12}” section below) before enrolling eligible participants into the trial. Written consent forms and audio-recordings of verbal consent will be stored securely at university sites.

### Additional consent provisions for collection and use of participant data and biological specimens {26b}

This trial does not involve collecting biological specimens.

## Interventions

### Explanation for the choice of comparators {6b}

Our pragmatic trial is designed to test whether the Community Navigator programme is an effective addition to routine care. Control group participants will also be offered a booklet with information about local community groups and social activities: this constitutes minimal signposting support with developing social connections and addressing loneliness, which could be provided within routine care, may be helpful to participants, and may mitigate feelings of disappointment for people allocated to the control group. Participants in both trial arms may access any other types of available support and treatment from the CMHT or other services: participation in the trial will not restrict access to routine care.

### Intervention description {11a}

#### The intervention

The Community Navigator programme was co-produced with service users and practitioners [7]. It is designed to increase social connections and reduce feelings of loneliness for people with TRD using secondary mental health services.

The programme offers participants up to 10 sessions of 1:1 support from a Community Navigator and attendance at three participant group “meet-ups” over a 6-month period. Participants are then offered one follow-up phone call and attendance at one more meet-up group during the next 2 months, so the intervention is concluded within 8 months from randomisation. Participants must attend at least three individual sessions with the Community Navigator for the intervention to be considered to be delivered per protocol. It is designed to be delivered primarily through face-to-face contact, but 1:1 and participant group “meet-ups” could all be arranged remotely if required to meet participants’ needs and preferences. To help develop social connections as part of the goals of the intervention, participants will be able to access modest financial re-imbursement to cover costs incurred (a maximum of £100 per participant). This will help ensure that poverty, or delays with personal budgets or other forms of statutory assistance, are not insuperable barriers to enacting goals to address loneliness. A detailed intervention manual was developed during the completed feasibility trial [7] and will guide the Community Navigators and supervisors in this trial.

The frequency and timing of 1:1 meetings can be decided flexibly between the participant and the Community Navigator, to meet the participant’s needs. Support will involve three stages:

- The Community Navigator will help people map their social world, using a previously developed mapping technique [16], recording people, places and activities which are subjectively important to the person, and considering lapsed activities or contacts which the participant might want to resume, or new social groups or connections they might wish to develop. This mapping will be combined with exercises helping people to review and develop their current and desired sense of belonging to social groups. These are derived from a social identities intervention, “Groups4Health”, developed for the general population [22] and were adapted for our TRD population with the support of the developers during the feasibility trial.
- This information is then used by the Community Navigator to help the participant develop a “personal connections plan”, recorded using a bespoke chart developed in the feasibility study [7]. Participants develop goals for increasing their social connections and are helped to break these goals down into steps, and identify personal resources and support within their network and community which can help them achieve their goals. Plans may involve engaging in new social groups and developing new contacts, or reconnecting with groups, friends or family; developing more meaningful social connections with people within existing social groups or activities that are valued and reinforce positive social identities.
- Next, the Community Navigator will provide different types of social support as appropriate to help participants enact their plans. This may include informational support (e.g., finding out about local groups or activities, or travel options to access them); practical support (e.g., using the budget; planning how a participant wants to introduce themselves in a social situation), or emotional support (e.g., going with a participant the first time to a new group, checking in with them after a phone call to a potential friend). Community Navigators will use solution-focused problem-solving approaches [23] to help participants break down goals into smaller steps if they are proving challenging. Participants’ social network maps will be reviewed and any progress in developing social connections encouraged and celebrated. Community Navigators will at all times help participants to develop social connections which are sustainable without long-term support from a Community Navigator; they will not provide befriending. For example, a Community Navigator would not typically accompany a participant several times to the same social group, as this support cannot be maintained after the end of the programme.

Group meet-ups will have an informal style and be lightly facilitated by the Community Navigators. They will encourage information-sharing about helpful local resources, experiences of developing connections and what has helped. Navigators will also introduce participants who may have shared interests or points of connection, and potentially help facilitate a meeting as part of 1:1 support if participants are keen. Responding to qualitative feedback from the completed feasibility trial, we plan a “soft ending” to the programme which does not feel abrupt. Navigators will offer a telephone or video call follow-up and one more group meet-up (the fourth of four available to each participant) after the ten 1:1 meetings, all to be completed before 8-month follow-up.

Participants will also continue to receive routine care from their mental health team (CMHT). If any treatment group participants are discharged from the CMHT more quickly than anticipated at trial enrolment, while they are still receiving support from a Community Navigator, this support will continue unless the clinical team explicitly direct that this is inadvisable. Local protocols will be developed for Community Navigators to be able to pass on any concerns about participants’ safety or wellbeing to the CMHT, even for participants who have been discharged from the team.

#### The control group

Participants in the control group will be offered written information about community resources and activities within their area. Participants will be encouraged to consider these resources themselves or discuss them with their care team. This constitutes a very low-cost, low-intensity comparator to the trial intervention, to test whether active, individualised support is superior to generic signposting. Participants will otherwise receive routine care from a CMHT, unaffected by their participation in the study. Routine care from a CMHT typically includes reviews by a psychiatrist and, regular meetings with a care coordinator, and may include psychological therapy.

### Criteria for discontinuing or modifying allocated interventions {11b}

The intervention is designed to be delivered flexibly, in a personalised way to meet individual participants’ needs and help them attain personal goals. Participants may choose to stop meeting their Community Navigator at any point during the intervention, and attendance at group “meet-ups” is also voluntary.

A participant may be withdrawn from trial treatment whenever continued participation is no longer in the participant’s best interests, but the reasons for doing so must be recorded.

Decisions to withdraw a participant from the trial intervention will be made ultimately by the site Principal Investigator, following consultation with the involved clinical team, and recorded in the participant’s case record form.

### Strategies to improve adherence to interventions {11c}

Community Navigators will use all available contact details held by the mental health team to contact participants. They will ask participants about their preferred contact methods and use these wherever possible. They will be as flexible as possible about the time and date of meetings and will seek to rearrange meetings in the event of participants cancelling or not attending meetings; keeping the invitation to participate open throughout the study intervention period.

In order to monitor intervention content and adherence, unblind members of the research team will collect brief information from Community Navigators about meetings with participants, which will be logged in the trial database. This will allow us to describe the number of sessions and meet-up groups attended by each participant and whether or not a participant was treated per protocol (at least three individual sessions).

### Relevant concomitant care permitted or prohibited during the trial {11d}

Routine care, whatever it involves for that individual, may continue to be provided to participants in the treatment and control groups for the duration of the study, without restriction.

### Provisions for post-trial care {30}

All participants may continue to receive routine care from their mental health team during and following the study. No other arrangements will be made to provide post-trial care.

Camden and Islington NHS Foundation Trust are the study sponsors and will provide NHS indemnity cover for any negligent harm to participants from taking part in the study.

### Outcomes {12}

All participants in the trial will be asked to complete self-report questionnaires at baseline, 8-month (end-of-treatment), and 14-month (6 months after end-of-treatment) follow-ups, and additional depression and loneliness ratings through phone or video calls at 4 and 11 months.

The primary outcome is depression symptom severity total score at 8 months end-of-treatment follow-up: measured by the Patient Health Questionnaire—PHQ-9 [20]. Secondary outcomes include measures of loneliness, depression, anxiety, personal recovery, multiple social identities, self-efficacy, self-sigma and social network size. For economic analyses, we will assess preference-based health-related quality of life using EuroQol EQ-5D 5 level (EQ-5D-5L) [24] and preference-based mental health-related quality of life using the Recovering Quality of Life (ReQoL) [25]. We will collect information about participants’ accommodation, employment status and use of mental and physical health services, using health records and an adapted CSRI measure [26]. The battery of measures for this trial was reviewed and refined following feedback from the feasibility trial and discussions with the Trial’s working group.

Participants will be asked to complete the following validated measures:

Screening measures collected at baseline:

- The Clinical Interview Schedule-Revised (CIS-R) [27] is a fully structured diagnostic instrument that was designed to be delivered by trained interviewers to assess minor psychiatric morbidity and generates diagnoses meeting ICD-10 criteria for depressive episodes.
- The DeJong Gierveld six-item Loneliness Scale (DJG-6) [28] is a 6-item, self-report measure of loneliness, yielding a total score and subscale scores for social and emotional loneliness
- Previous anti-depressant use

#### Primary outcome

- Depression severity measured using the Patient Health Questionnaire (PHQ-9) [20], a nine-item measure of depression symptom severity: total score at 8 months end-of-treatment follow-up PHQ-9.\

#### Secondary outcomes

- We will also report PHQ-9 total score data at 4-, 11- and 14-month follow-up points.
- We will create two further variables from PHQ-9 scores for analysis at 8 and 14 months follow-up points: (a) recovery from depression, where PHQ-9 score will be dichotomised for analysis, where ≥ 10 is the clinical cut off for depression [20]; and (b) Substantial improvement in depression: a dichotomous variable for whether or not a reduction in PHQ-9 score since baseline of at least 5 points has been achieved, based on the established threshold for reliable and clinically significant change [29].
- The UCLA Loneliness scale (ULS-8) [30] is an 8-item scale measuring loneliness which has good established psychometric properties and has been used previously in mental health populations (all assessment points).
- The Generalised Anxiety Disorders Scale (GAD-7) [31] is a seven-item self-report measure of anxiety (baseline, 8 months follow-up, 14 months follow-up).
- The Questionnaire on the Process of Recovery (QPR) [32] is a 15-item self-report measure of personal recovery (baseline, 8 months follow-up, 14 months follow-up).
- The Multiple Identities Scale (MIS) [33] is a four-item self-report scale measuring multiple social identities (baseline, 8 months follow-up, 14 months follow-up).
- The Brief Rosenberg self-esteem scale (B-RSES) [34] is a 5-item scale measuring self-esteem (baseline, 8 months follow-up, 14 months follow-up).
- The Discrimination and Stigma Scale (DISC-12 subscale) [35] is an interview-based scale which measures experiences of mental health-related discrimination in key areas of everyday life and social participation, including work, marriage, parenting, housing, leisure and religious activities. To measure self-stigma, we will be using the four-item self-stopping behaviours subscale from the Discrimination and Stigma Scale (baseline, 8 months follow-up, 14 months follow-up).
- Lubben Social Network Schedule (LSNS-6) [36] is one of the most widely used questionnaires to quantitatively assess social network size and we will use the six-item Lubben Social Network Schedule to measure social network size (baseline, 8 months follow-up, 14 months follow-up).
- The EQ-5D-5L [24] is a five-item self-report health outcome measure (baseline, 8 months follow-up, 14 months follow-up).
- The Recovering quality of life (ReQoL) [25] is a 10-item self-report measure of quality of life developed for use across all mental health populations (baseline, 8 months follow-up, 14 months follow-up).
- Client Service receipt Inventory (CSRI) [26] is a tool used to collect information on the range of services and supports participants may use (baseline, 8 months follow-up, 14 months follow-up).
- Daytime Activities Questionnaire (all assessment points).
- The Credibility and Expectancy questionnaire [37] is a six-item scale that measures treatment expectancy and credibility. We will use a single-item adapted version (Baseline).

The secondary outcome for substantial improvement in depression, using PHQ-9 data, was revised in an amendment to the original approved protocol to a change from baseline of at least 5 points, reflecting guidance in previous literature. This change was made prior to the statistical analysis plan being finalised and before the database lock. Please see Additional file 1: Appendix 1 for further information about all amendments to the trial protocol.

### Participant timeline {13}

Table 1 shows the schedule of assessments.

**Table 1:** Schedule of assessments

	**Screening**	**Treatment phase**		**Follow-up**	**Final visit**
**Visit no:**	1	2	3	4	5
	Screening and baseline assessment	4-month follow-up	8-month end-of-treatment follow-up	11-month follow-up	14-month (6 months post-treatment) follow-up
**Window of flexibility for timing of visits:**	n/a	+ 2 months	+ 3 months	+ 2 months	+ 3 months
**Informed consent**	YES				
**Eligibility confirmation (CIS-R and DJG measures)**	YES				
**Intervention credibility measure**	YES				
**All outcome measures and service user information listed in s. 10.1**	YES		YES		YES
**Depression (PHQ-9) and Loneliness (ULS-8) measures only**	YES	YES		YES	
**Randomisation**	YES				
**Trial intervention/treatment**		YES	YES		
**Adverse events active monitoring**	YES	YES	YES		
**Adverse events review**	YES	YES	YES	YES	
**Withdrawal**	YES	YES	YES	YES	YES

### Sample size {14}

We will recruit 306 participants. The standard calculation to detect a 0.4 standard deviation (sd) difference in PHQ-9 depression score between arms with 90% power and 5% alpha requires 132 participants per arm. Adjusting for baseline PHQ reduces the sample size by *r*-squared, where *r* is the correlation between baseline and follow-up outcome values [38]. Assuming a correlation of 0.5 and 15% attrition (informed by the feasibility trial [7]) results in a sample size of 117 participants per arm. Assuming four sites, three navigators per site, and an ICC for clustering by navigator of 0.05, results in a design effect of 1.6 (based on 13 participants per navigator). Inflating the sample size for clustering in the intervention arm only (to 188), then adjusting group sizes to be equal, produces a sample size of 153 participants per arm.

In response to peer review comments, we have checked the sample size calculation using simulation and have found that we do obtain 90% power, as calculated, when analysing the data using a mixed model with ML, treating each control subject as a cluster of size 1. However, as noted by the reviewer, the confidence interval coverage and type I error are slightly improved by analysing the data using REML with the Kenward-Rogers adjustment. This results in a slight loss of power but is the analysis we now intend to use. The power to detect a (standardised) difference of 0.4 is reduced to 87%, or equivalently, we have 90% power to detect a difference of 0.42. This still provides 90% power to detect a minimum personally meaningful difference on PHQ-9 between groups of about 2 points [39].

### Recruitment {15}

Study researchers will regularly visit participating clinical teams, attend team meetings and arrange individual meetings with clinicians. We will seek help from all involved clinicians to identify potentially eligible participants, let them know about the study and, if they are interested, ask if a researcher may contact them. Local researchers within the involved health organisation will also screen team caseloads and contact potentially eligible participants. A leaflet about the research study, designed by the trial Lived Experience Advisory Panel, was ethically approved for distribution to potential participants. Study researchers will use all contact details provided to contact potential participants, and offer as many meetings or as much time as a participant requires to understand the study and decide if they want to take part.

Additional strategies to help with participant recruitment were implemented following ethical approval for an amendment to the original trial protocol. Please see Additional file 1: Appedix 1 for details of all protocol amendments:

1. A poster and revised leaflet for potential participants, inviting people to either contact their clinical team or contact the study researchers directly for more information about the study if they wish
2. Permission to display leaflets and posters in other local services (e.g. local day centres) where they may be seen by potential participants
3. Permission for study researchers to be present at clinic waiting rooms (with clinicians’ agreement) to be on hand to talk to potential participants who would like to hear more about the study
4. Permission to send group mail-outs with information about the study to service users, where consistent with local services’ consent to contact procedures.

## Assignment of interventions: allocation

### Sequence generation {16a}

Following screening and baseline assessments, eligible participants will be randomised to a treatment group or a control group. We will use block randomisation stratified by site. We will use a web-based randomisation and clinical data management system for recording CRF data (Red Pill), which is provided by a company called Sealed Envelope Ltd. Sealed Envelope has been assessed by PRIMENT Clinical Trials Unit (CTU) to ensure that adequate processes are in place and are being followed for quality management, software development and data security purposes. The Red Pill service has been inspected by MHRA for GCP compliance, and there is a Master agreement and Data Processing agreement in place between UCL and Sealed Envelope to ensure compliance and agreement with clinical trial regulations and data protection laws. Any data that is collected that contains Personal Identifiers such as name, address and NHS numbers will be stored in UCL Data Safe Haven which has been certified to the ISO27001 information security standard and conforms to NHS Digital’s Information Governance Toolkit. Any data that is to be shared outside of UCL will be anonymised and PRIMENT CTU guidance on data sharing will be followed.

### Concealment mechanism {16b}

Only non-blind members of the trial team will be set up with investigator accounts on the trial Sealed Envelope Red Pill database, which makes participants’ allocation status visible and generates email notifications of new randomisations. Blinded study researchers will not be given this investigator access and thus be unable to see participants’ allocation status on the trial database. Randomisations are generated by an algorithm in the Sealed Envelope Red Pill system, so none of the study team have any knowledge of future allocations. Likewise, block sizes for randomisations are known only to the Sealed Envelope system and the lead statistician.

### Implementation {16c}

Group allocation will be generated through Sealed Envelope by the non-blind Trial Manager or trial administrator who will then communicate to participants, clinical teams and Community Navigators the group allocations.

## Assignment of interventions: blinding

### Who will be blinded {17a}

Participants and care providers cannot and will not be blind to participants’ allocation status. Study researchers will be blinded to participants’ allocation status. For data analysis, groups will be labelled only as 1 and 2: the study statistician and health economist who analyse the trial data will be blind to which group is which. The trial manager, trial administrator, peer researcher and the Chief investigator will not be blinded. They will let participants know their allocation status; oversee reporting of serious adverse events, collect process data from the Community Navigators and conduct qualitative interviews for the qualitative sub-study. These researchers will not conduct follow-up interviews with participants, to ensure the researcher-blind integrity of outcomes data.

### Procedure for unblinding if needed {17b}

On request to the Clinical Trials Unit, data regarding participants’ characteristics and baseline scores on outcome measures can be downloaded from the secure trial database, identified only as Groups 1 and 2, in order to check participant characteristics, data completeness and the balance achieved through randomisation while maintaining blinding. We will follow advice from the Data Monitoring and Ethics Committee and on their advice, the senior trial statistician could be unblinded to look at differences in adverse events or outcomes between groups. The blinded study researchers will ask participants not to reveal whether or not they were allocated to get support from a Community Navigator, but it is possible some participants may reveal their allocation status to the researcher nevertheless. The research team will keep a log of such unblinding incidents and, wherever possible, an alternative researcher who is still blinded will carry out follow-up assessments.

## Data collection and management

### Plans for assessment and collection of outcomes {18a}

Eligibility screening, baseline and follow-up data collection at 8-month end-of-treatment and 14-month follow-ups may be conducted through in-person meetings, video calls or phone calls, as the participant prefers. Four-month and 11-month data will be collected through a self-report online form or phone or video call only. The trial administrator or trial manager will check screening forms to confirm eligibility criteria are met before initiating randomisation through the trial electronic randomisation database. Checks of data completeness and accuracy will be made periodically by the Trial Manager or administrator, supported by the Clinical Trials Unit.

All study researchers will be trained in Good Clinical Practice (GCP) and will receive bespoke training from the Trial Manager and senior study researchers in conducting participant recruitment, including assessing capacity to consent, and study data collection measures. This will involve role play practice using measures with members of the study Lived Experience Advisory Group.

Data collection Case Record Forms are available from the corresponding author on reasonable request.

### Plans to promote participant retention and complete follow-up {18b}

Participants’ decisions to withdraw from (a) the study intervention and (b) the trial will be recorded separately. Where a participant decides to disengage from seeing the Community Navigator before the scheduled end of the programme, the study research team will seek permission to continue to contact the participant to collect outcomes data at follow-up points. If a participant asks to withdraw from the research study, the research team will clarify whether the person (a) just wants no more contact from the researchers, or (b) wishes to withdraw their participation completely, including deleting any data previously collected. If the participant just wants no further contact with the researchers, the study team will retain data collected up to that point and collect data from patient records in accordance with the person’s consent.

Overdue follow-ups will be tracked using the study database. In the case of overdue follow-up visits, reasonable attempts will be made to contact the participant. These will include trying all contact routes provided by the participant and contacting the referring clinician.

### Data management {19}

Paper Case Report Forms (CRF) and patient notes will be stored at research sites. Electronic CRFs will be stored in Red Pill/Sealed Envelope, a GCP compliant database used by UCL’s Priment Clinical Trials Unit. Data entry will only be carried out following training on the secure, password-protected database. Trial data will be coded at the entry stage using predefined structures. Back-ups will be made of the trial database on a monthly basis.

Checks of data entry and eligibility are carried out at baseline for all participants prior to randomisation. Data will also be validated with source data verification (first 5 participants at each site, and 10% of all participants), database checks and statistician’s checks. Queries and missing data will be monitored, with the aim of resolution within a month. At sites with a high level of discrepancy, staff will be retrained. Delivery of the intervention will be monitored for adherence to protocol and correct timing of intervention using session log records.

Once all data have been received and cleaned, the database will be locked for analysis, without the possibility of further edits. At the end of the trial, all essential documentation will be archived securely for a minimum of 10 years. In addition to this data management summary, the trial team has also developed detailed guidance for sites (plans on Data Management, Monitoring, Source Data Verification, and a database guidance document) which are stored in the Trial Master File and Investigator Site Files.

### Confidentiality {27}

Referral forms for named, potential participants who have agreed to be referred to the study and Identifiable information about trial participants will all be stored in the University College London Data Safe Haven, a highly secure data store with access limited to essential study researchers. A key linking participant names and ID numbers will also be stored in the Data Safe Haven.

All data collected about participants, including personal data such as demographic characteristics, will be stored in the trial electronic “Sealed Envelope” database where participants are identified only by their trial ID number. Paper copies of data collection forms will similarly be identified only by participant ID and will be stored securely in university premises.

### Plans for collection, laboratory evaluation and storage of biological specimens for genetic or molecular analysis in this trial/future use {33}

This trial does not involve collection or storage of biological specimens.

## Statistical methods

### Statistical methods for primary and secondary outcomes {20a}

A Statistical Analysis Plan will be finalised and approved prior to commencing analysis. The analyses and subsequent reporting will be guided by the CONSORT recommendations [40]. Analyses will be performed on a modified intention to treat basis, in which participants will be analysed according to their allocated group using all available data for a given outcome and time-point. A CONSORT diagram will be presented to provide a detailed description of participant numbers at each time-point during the trial.

The baseline demographic and clinical characteristics of the participants will be presented in a table summarised separately by study arm. Categorical variables will be reported as counts and percentages. Continuous variables will be summarised as either means and standard deviations (SD) or medians and interquartile ranges, depending on the distribution of the data. No statistical tests will be performed to assess baseline differences between study arms. Any notable imbalances may lead to additional adjusted sensitivity analyses.

#### Primary outcome statistical analysis

The primary analysis of the PHQ-9 score at 8 months follow-up (end-of-treatment) comparing intervention and control groups will use a mixed model (estimated using REML, with the Kenward-Roger adjustment) to perform an individual-level analysis and will follow guidance [41] in adjusting for navigator clustering in the intervention arm only (random coefficient model): specifically, each control subject will be treated as a cluster of size 1. This model will also adjust for baseline PHQ-9 score and site using fixed effects. The estimated intervention effect will be reported with a 95% confidence interval and *p*-value. This analysis will use available data only. All modelling assumptions will be checked. In particular, a confirmatory analysis will be performed using the heteroscedastic model [41] which allows the residual variance for intervention and control groups to differ. Withdrawals from the study, loss to follow-up and other missing outcome data will be summarised separately by randomised group. The primary analysis will be a complete case analysis. However, we will investigate whether there are any predictors of missingness. If any are found, these will be included in an adjusted analysis as a supportive analysis.

#### Secondary outcome statistical analysis

The effect of the intervention on secondary outcomes will be assessed using analogous methods to those used for the primary outcome. Most of the secondary outcomes are numerical and hence will be analysed using a similar model to that used for the primary outcome, whereas the binary outcomes (those derived from PHQ-9) will be analysed using a mixed-effects logistic regression model. *P*-values will not be reported for secondary analyses. The number and type of adverse events will be summarised by study arm.

#### Health economics analysis

##### Measurement of costs and outcomes

We will calculate the incremental cost per quality-adjusted life year (QALY) gained of Community Navigators plus routine care compared to routine care over 14 months from a health and social care cost perspective, using the EQ-5D-5L to calculate QALYs. Secondary analyses will include (i) using the ReQoL to calculate QALYs; (ii) including wider societal costs such as productivity and absenteeism and use of voluntary services.

We will calculate the cost of delivering the Community Navigator intervention, including training and supervision based on activity reported by the Community Navigators. Unit costs for staff costs and Community Navigator costs will be taken from the Personal Social Services Resource Unit (PSSRU) [42]. We will collect all participants’ mental health service use information from health records, including contacts with community mental health staff and any use of inpatient or crisis services. Other health and social care resource use including primary care will be collected from an adapted CSRI at completed with researcher support at baseline, 8 months and 14 months asking about the past 6 months. The feasibility trial [7] identified that our TRD client group also had a range of physical health needs, so we will ask about planned and unplanned acute hospital resource use. Participants will be asked to report use of social prescribing schemes, befriending, peer support groups and other social clubs, organisation and voluntary sector groups as part of a bespoke questionnaire at baseline and each follow-up time-point to collect detailed information on any additional activities that might have occurred as a result of the intervention, with equivalent information also collected for the arm receiving only routine care. Health and social care resource use will be costed using NHS reference costs [43] and PSSRU [42]. For wider societal costs, we will ask about current employment status and for employed participants’ time off work sick in the past 6 months, with additional absenteeism and presenteeism information collected using the Work Productivity Activity Impairment Questionnaire [44], incorporated into the CSRI. This will be costed using the human capital approach. Costs of voluntary services and accommodation will also be included in wider societal costs.

QALYS will be calculated using the EQ-5D-5L and appropriate UK tariff as the area under the curve adjusting for baseline [45]. EQ-5D-5L has been shown to be a valid and responsive measure for depression [46] although there is limited evidence for TRD. The use of the EQ-5D-5L is recommended in the National Institute for Health and Care Excellence [47] allowing for comparisons across disease areas. In the Community Navigators feasibility study [7] (noting very small patient numbers), patients in the intervention group had a 0.19 increase in utility compared to 0.05 in the control group, indicated that the EQ-5D-5L may be sensitive to a treatment effect. However, to address potential limitations with the EQ-5D-5L, we will also calculate QALYs using the ReQoL and its published tariff, a measure of mental health-related quality of life.

We will report the mean incremental cost per QALY gained of the Community Navigator programme plus routine care, compared to routine care, over 14 months for all analyses. The denominator and numerator of the incremental cost per QALY gained will be calculated using regression analysis, accounting for baseline, site as a fixed effect and Community Navigator as a random effect. Other potential coefficients for inclusion will be considered as part of a health economics analysis plan signed off before database close. 95% confidence intervals, cost-effectiveness planes and cost-effectiveness acceptability curves will be calculated based on bootstrapped results. Assuming missing at random, we will use multiple imputation using chained equations to account for missing data. We will consider any potential missing not at random effects and sensitivity analyses for any uncertainty. All costs and consequences beyond 12 months will be discounted at a rate of 3.5% in line with NICE guidance.

### Interim analyses {21b}

Trial recruitment and intervention engagement will be monitored and continuation criteria reviewed during the internal pilot phase of the trial. No interim analyses are planned.

### Methods for additional analyses (e.g., subgroup analyses) {20b}

Several additional analyses are planned. These include (1) an analysis to adjust for any baseline imbalance caused either by chance or missing data; (2) an analysis that includes PHQ-9 data from all five time-points (including baseline) using a 3-level mixed model; (3) an analysis to explore the mediating effect of loneliness on depression across the five time-points (main results permitting); (4) an analysis to explore the effect of baseline expectations and credibility of the intervention on the outcome (main results permitting). There are no planned subgroup analyses.

### Methods in analysis to handle protocol non-adherence and any statistical methods to handle missing data {20c}

Adherence to intervention will be described, e.g., in terms of the mean (SD) number of sessions attended. In addition, a complier average causal effect (CACE) analysis may be performed to adjust for any non-adherence to the intervention, where adherence to the intervention is defined as attendance at three or more meetings with Navigators. A further analysis may investigate whether there is a dose response effect of the number of Navigator meetings.

Withdrawals from the study, loss to follow-up and other missing outcome data will be summarised separately by randomised group. Potential bias due to missing data will be investigated by comparing the baseline characteristics of participants with and without missing values. Depending on the quantity of missing values, predictors of missingness may be identified. We will then perform a sensitivity analysis that includes these predictors of missingness as covariates in the primary analysis model. Various multiple imputation strategies may also be performed, if deemed appropriate, assuming either “missing at random” (MAR) or “missing not at random” (MNAR).

### Plans to give access to the full protocol, participant-level data and statistical code {31c}

The full trial protocol is publicly available on the funders’ website: Randomised controlled trial of the Community Navigator programme to reduce loneliness and depression for adults with treatment-resistant depression in secondary mental health services—NIHR Funding and Awards.

All requests for the participant-level dataset and statistical code should be submitted to the corresponding author for consideration. Access to anonymised data may be granted following review, involving the trial statistician and Priment Clinical Trials Unit team.

## Oversight and monitoring

### Composition of the coordinating centre and trial steering committee {5d}

Day to day management of the trial is overseen by the Trial Management Group (TMG), which consists of all the study co-Investigators and the trial manager and is chaired by the Chief Investigator. The TMG meets monthly. Study researchers and a representative of the Lived Experience Advisory Panel also attend TMG meetings.

Advice and monitoring of study documents, databases and procedures is provided by a team from the Priment Clinical Trials Unit, who support the study and undertake oversight responsibilities for quality assurance, delegated by the sponsor.

The study Lived Experience Advisory Panel (LEAP) consists of nine people with personal experience of depression or anxiety and using mental health services, who bring expertise by experience. The LEAP meets regularly, and LEAP members contribute to developing study documents and recruitment materials, recruiting and training Community Navigators, training study researchers, analysing qualitative data and contributing to writing up and disseminating study findings in due course. The LEAP group is supported and coordinated by the study peer researcher and co-Investigators from the McPin Foundation, a not-for-profit organisation which promotes service user involvement in research. The LEAP has no formal oversight responsibilities for the trial but provides additional scrutiny of trial documents and processes from a lived experience perspective.

The Independent Trial Steering Committee (TSC) has six members, including a clinical academic with extensive trials experience, an academic loneliness expert, two lived experience members both of whom bring experience of mental health practitioner roles, and an academic statistician and a health economist. The TSC meets at least annually. Approved minutes from TSC meetings and TSC recommendations are reported to the sponsors and the funders.

### Composition of the data monitoring committee, its role and reporting structure {21a}

The Data Monitoring and Ethics Committee (DMEC) has four members: an academic with expertise in TRD research and substantial trials experience, a senior academic psychiatrist, a statistician and a lived experience researcher who has a senior academic role. The DMEC has a specific remit to scrutinise participant safety and protocol breaches and can ask to see data sets and unblind data reports if required for these tasks. The DMEC meets at least annually and reports to the Chair of the TSC.

### Adverse event reporting and harms {22}

Serious adverse events (SAEs) are defined as any event which results in death, is life-threatening, requires hospitalisation or prolongation of existing hospitalisation, or results in persistent or significant disability or incapacity. SAEs will be monitored by researchers through a structured safety monitoring form completed at each follow-up assessment, and through feedback from Community Navigators, participants or involved clinicians at trial sites. Each SAE will be recorded on a case record form. The site Principal Investigator will liaise with the relevant clinical team and the participant if required to assess the event’s seriousness, expectedness and study-relatedness. SAE reports will be sent to the Clinical Trials Unit (delegated by the sponsor) and the DMEC Chair and clinical academic for review. Any serious, unexpected, study-related adverse events (SUSARs) will also be reported to the ethics committee within 7 days of the Chief Investigator becoming aware of them.

Any study-related harms which do not constitute SAEs will also be recorded using CRFs. The study team will also keep a log of any negative feedback or events relating to participants’ involvement in the study, to inform any needs for changes to trial processes or researcher training. These non-serious events will also be reported to the DMEC meetings for external review.

### Frequency and plans for auditing trial conduct {23}

Formal audits of trial procedures and data management may be conducted by the sponsor or the Clinical Trials Unit on behalf of the sponsor at any point during the study.

### Plans for communicating important protocol amendments to relevant parties (e.g. trial participants, ethical committees) {25}

Any proposed changes to the trial protocol will be reviewed and approved by the Clinical Trials Unit, the sponsor and the funders, then submitted for approval from the Research Ethics Committee (REC). No protocol amendments will be implemented until REC approval and site approval are then confirmed.

The funders will be informed of new protocol versions so an up-to-date version of the protocol is available on the funders’ website.

### Dissemination plans {31a}

This study is funded by the English National Institute for Health and Care Research, Health Technology Assessment Programme (NIHR HTA). It has been added to the NIHR Clinical Research Network Portfolio and included on the ISRCTN registry.

Our study will provide high-quality evidence about the effectiveness of a novel programme to address loneliness and social isolation and reduce depression for people with TRD in secondary care. We will provide a final report for the funders in adherence to their requirements. We will seek to publish scientific papers in open access journals, reporting the trial protocol, main results, qualitative investigations and health economics findings. In collaboration with the trial Lived Experience Advisory Panel members, we will co-produce a briefing document for policy makers, a guide to support future programme delivery in mental healthcare settings, and articles in publications read by practitioners and commissioners and by service users and the public. We will present findings at stakeholder workshops, meetings or conferences. We will update the Community Navigator intervention manual and training manual and make these publicly available, to speed up knowledge transfer if warranted by trial findings.

The Study CI will draft a publications plan identifying proposed lead and included authors and timescales for publications. This will be discussed and agreed at study TMG meetings. Authorship will be based on ICMJE guidelines. All proposed publications will be discussed with and reviewed by Priment CTU prior to publishing.

Trial participants will be asked during the consent process if they would like to receive a lay summary of the study findings. The study team will work with the trial Lived Experience Advisory Panel to develop an accessible summary of the study findings to send to all interested participants when the study is over.

## Discussion

Our trial addresses a gap in evidence regarding theory-driven and potentially effective social interventions for people with TRD that, by targeting loneliness and social isolation, would act via a different mechanism from the pharmacological and psychological treatments currently available. Our intervention could help mental health services provide genuinely biopsychosocial care for an important clinical group who are not adequately helped by current treatments.

Research on interventions for loneliness in mental health is in its infancy: effective models of support have yet to be established [17]. The Community Navigator programme represents one socially focused approach to addressing loneliness, involving employing additional staff with a bespoke role solely to help people with social connections, in addition to usual care. Other studies have developed and tested social interventions with different mental health client groups which involve seeking to train and upskill the existing care team [48]. A range of psychological interventions for loneliness has also been developed [49]. Whatever the result of our trial, further research is required to determine the most acceptable and effective intervention models to reduce loneliness and improve health and social outcomes across a range of clinical groups. To our knowledge however, the Community Navigator trial is the first study of a programme designed to reduce depression through alleviating loneliness for people with TRD in a secondary care context. Findings will provide rich information to inform practice and future research.

## Trial status

The current trial protocol is Version 4.0, 7th July 2023. The first trial participant was recruited on 7th September 2022. Participant recruitment is currently due to be completed by April 2024.

### Supplementary Information

**Additional file 1: Appendix 1.** Summary of protocol amendments. **Appendix 2.** Participant Consent Form. **Appendix 3.** Participant Information Sheet.

## Data Availability

The Community Navigator programme intervention manual and training manual will be made publicly available on the study website at the need of the trial. All requests for the participant-level dataset and statistical code should be submitted to the corresponding author for consideration. Access to anonymised data may be granted following review, involving the trial statistician and Priment Clinical Trials Unit team.

## Abbreviations

CMHT: Community Mental Health Team
CIS-R: Clinical Interview Schedule-Revised
CRF: Case report form
CRN: Clinical Research Network
CSRI: Client Service Receipt Inventory
DJG-6: 6-Item De Jong Gierveld Loneliness Scale
DISC: Discrimination and Stigma Scale
DMEC: Data Monitoring and Ethics Committee
EQ-5D-5L: EuroQol EQ-5D 5 level (EQ-5D-5L)
EU: European Union
GAD-7: General Anxiety Disorder Assessment
GAfREC: Governance Arrangements for NHS Research Ethics
GCP: Good Clinical Practice
GDPR: General Data Protection Regulation
HRA: Health Research Authority
ICD-10: International Classification of Diseases 10th Revision
ISRCTN: International Standard Randomised Controlled Trial Number
LEAP: Lived Experience Advisory Panel
LSNS-6: Six-item Lubben Social Network Schedule
MIS: Four-item Multiple Identities Scale
NIHR HTA: National Institute of Health Research Health Technologies Assessment Programme
PHQ-9: Patient Health Questionnaire – Mental Health Disorders
QALY: Quality-adjusted life year
QPR: Questionnaire on the Process of Recovery
RCT: Randomised controlled trial
REC: Research Ethics Committee
REQOL: Recovering Quality of Life
B-RSES: Brief Rosenberg self—esteem scale
SAE: Serious adverse event
SDV: Source Document Verification
SOP: Standard Operating Procedure
SUSAR: Suspected unexpected serious adverse reaction
TMG: Trial Management Group
TRD: Treatment-resistant depression
TSC: Trial Steering Committee
ULS-8: University Of California, Los Angeles Loneliness Scale
