# Supplementary material for: Randomised controlled trial of the Community Navigator programme to reduce loneliness and depression for adults with treatment-resistant depression in secondary community mental health services: trial protocol
Source: Trials. 2023 Oct 6;24:652. doi: 10.1186/s13063-023-07684-4 (PMC10559405; doi:10.1186/s13063-023-07684-4)
Supplement: Supplementary file 1 — Additional file 1: Appendix 1. Summary of protocol amendments. Appendix 2. Participant Consent Form. Appendix 3. Participant Information Sheet. [file 13063_2023_7684_MOESM1_ESM.docx]

**Appendix 1. Summary of Protocol Amendments**

| **Amendment type** | **Date** | **Protocol Version** | **Change(s)** | **Reason for the change** |
| --- | --- | --- | --- | --- |
| Substantial | 09-NOV-2022 | 2 | Addition of study promotion materials (posters and updated leaflets) and additional recruitment approaches (publicising the study in other mental health services accessed by potential participants) | To improve recruitment and add alternative options to promote the study |
| Non-substantial | 15-MAY-2023 | 3 | Rewording of software used for online survey | UCL changed the platform used for online surveys and the previous version of the protocol named the old platform |
| Substantial | 07-JUL-2023 | 4 | - Dementia (F00-F03) and Mild Cognitive Impairment (F06.7) added as exclusion criteria | - New sites joining led to older adults’ teams being a part of the study. The TMG and older adults’ researchers advised that explicit dementia exclusion criteria be added to ensure the trial sample remains a clearly defined TRD clinical group. |
|  |  |  | - Detail added on the use of a the PHQ-9 as a secondary outcome | - The proposed secondary outcomes from the PHQ-9 measure were specified in more detail, to align with the trial statistics analysis plan which has now been drafted. |

**[
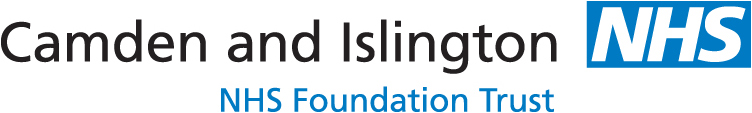
](https://www.google.co.uk/url?sa=i&rct=j&q=&esrc=s&source=images&cd=&cad=rja&uact=8&ved=0ahUKEwjnhOSIw5jYAhXGDcAKHYrvAhgQjRwIBw&url=https://www.whatdotheyknow.com/request/240634/response/593860/attach/html/7/FOI%20Acknowledgement%202014%20166.pdf.html&psig=AOvVaw0Uit3hSwkeP6cGe5Hj71xM&ust=1513857022566159)Appendix 2. Participant Consent Form**

[HEADER: SITE LOGO]

**Participant Consent Form**

**COMMUNITY NAVIGATOR TRIAL**

**Study Title: A Randomised controlled trial of the Community Navigator programme to reduce loneliness and depression for adults with treatment resistant depression in secondary mental health services**

Site Name:

|  | Please initial box |
| --- | --- |
| 1. I confirm that I have read the information sheet dated 22 March 2022, v2.0 about the above study. I have had the opportunity to consider the information, ask questions and have had these answered satisfactorily. |  |
| 1. I understand that my participation is voluntary and that I am free to withdraw at any time without needing to give a reason, and this would not affect my medical care or legal rights. |  |
| 1. I understand that my GP/ Community Mental Health Team will be informed of my participation in the study. I understand that if I am allocated to receive support from a Community Navigator, a record of their meetings with me will be kept in my patient notes. |  |
| 1. I understand that the information collected about me may be used to support other research in the future and may be shared anonymously with other researchers. |  |
| 1. I understand that relevant sections of my medical notes and data collected during the study, may be looked at by individuals from the trial team, from regulatory authorities or from the NHS Trust, where it is relevant to my taking part in this research. I give permission for these individuals to have access to my mental health records and my GP records. |  |
| 1. I agree that a researcher may contact me at subsequent points during this year, to see whether I am willing to take part in other data collection activities. I am aware that when they contact me, I can decide whether to take part or not. |  |
| 1. I consent to the information collected about me for this study being stored securely at the researcher’s work place (University College London, University of Birmingham or University of York). |  |
| 1. I understand that I will be offered a £20 gift voucher for my participation in the research interview, once I have taken part in it. |  |
| 1. I understand that the information held and maintained by the Health and Social Care Information Centre and other central UK NHS bodies may be used to help contact me or provide information about my health status. |  |
| 1. I agree to take part in the above study. |  |
| 1. (For video-call or phone consent only) I agree to give my consent through a video-call or phone call, consenting to statements read out by the study researcher. I understand my consent will be audio-recorded and stored by the researchers. |  |
| 1. **Optional:** In the event of the research team having difficulties contacting me directly for the study data collection activities, I consent to a researcher contacting a friend or a family member or a member of staff if I have named them below. | Yes/No |
| 1. **Optional:** I consent to a researcher contacting me to invite me to complete an online survey about my experience of the recruitment process | Yes/No |

**My preferred contact details:**

Name:

Address:

Phone number(s):

E-mail address:

I consent to being contacted by:

Phone call

Text

E-mail

Letter

Preferred method of contact:

Phone

E-mail

Letter

Is there anything else we need to know about communicating with you? e.g large print, hearing impaired, speaking slowly, calling from withheld numbers?

**Contact details of friends, family members or carers I am happy for a researcher to contact if necessary to contact me for a follow-up interview:**

*(If possible, please provide details of any friends, family members or carers whom researchers could contact if unable to contact you directly for a follow-up interview.)*

|  | Personal contact #1 | Personal contact #2 |
| --- | --- | --- |
| Name |  |  |
| Relationship to participant |  |  |
| Address |  |  |
| Phone number(s) |  |  |
| email |  |  |

**Contact details of mental health staff working with me I am happy for a researcher to contact if necessary to contact me for a follow-up interview:**

*(If possible, please provide details of any mental health staff whom researchers could contact if unable to contact you directly for a follow-up interview.)*

Name:

Job title:

Service:

Contact details (if known)

I would like a copy of a report with the study findings when the study is over:

Yes

No

Please sign this consent form below to confirm your consent to take part in the study.

Name of Participant Date Signature

Name of Person Date Signature

taking consent

When completed: 1 for participant; 1 for researcher site file; 1 (original) to be kept in medical notes.

**[
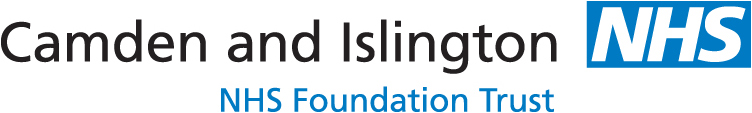
](https://www.google.co.uk/url?sa=i&rct=j&q=&esrc=s&source=images&cd=&cad=rja&uact=8&ved=0ahUKEwjnhOSIw5jYAhXGDcAKHYrvAhgQjRwIBw&url=https://www.whatdotheyknow.com/request/240634/response/593860/attach/html/7/FOI%20Acknowledgement%202014%20166.pdf.html&psig=AOvVaw0Uit3hSwkeP6cGe5Hj71xM&ust=1513857022566159)Appendix 3. Participant Information Sheet**

[HEADER: SITE LOGO & SPONSOR LOGO]

**Participant Information Sheet**

**COMMUNITY NAVIGATOR STUDY**

## **Study Title: A randomised controlled trial of the Community Navigator programme to reduce loneliness and depression for adults with treatment-resistant depression in secondary mental health services**

**Invitation to participate in a research study**

You are being invited to take part in a research study. Before you decide whether to take part it is important for you to understand why the research is being done and what it will involve. Please take time to read the following information carefully and discuss it with others if you wish. Ask us if there is anything that is not clear, or if you would like more information.

**What is the purpose of the study?**

We have developed and tested a programme of support to improve community connections for people who are using mental health services, and who are experiencing long-term depression and feelings of loneliness. A Community Navigator helps people to consider their current social contacts and social activities and then to develop a plan to engage with desired activities and begin building or strengthening relationships within their local community. The Community Navigators will also be running sessions where participants can meet, which people will be invited to attend. In this study, we will test whether support from a Community Navigator helps people to feel more connected to their social communities and less lonely, leading to an improvement in their wellbeing and quality of life.

**Why have I been asked to take part?**

You have been asked to take part because you have been using one of the secondary mental health services participating in this study. You have also indicated to a member of staff in the service which has been supporting you that you were happy to discuss participating in the study with a researcher.

**Do I have to take part?**

It is completely up to you to decide whether or not you would like to take part. If you do not feel the study is relevant to you or you do not wish to increase your social connections, then you do not need to take part. If you decide to take part, you will be given this information sheet and asked to sign a consent form. If you do decide to take part, you are still free to withdraw at any time and without giving a reason. A decision to withdraw at any time, or a decision not to take part, will not affect the standard of care you receive from mental health services now or in the future. If a researcher is unable to contact you for a follow-up interview, or if you decide you do not want to take part in a follow-up interview, the research team will continue to use information you have already provided and information from your patient records, unless you tell the research team or mental health staff involved in your care that you do not want this to happen.

**What will happen if I take part?**

Before your invitation to take part in the study is confirmed, a researcher will complete a brief questionnaire with you about your mood and how connected to others you feel. To make sure the study only includes people who are currently experiencing a certain level of depression and some degree of loneliness. only people whose answers score in a certain range on these measures will be included in the study. If you are not offered the chance to take part in the study following these brief questionnaires, the researcher will offer to send you some information about local community resources which may help with developing connections with others.

If you agree to take part in the study, you will be randomised into one of two groups, Group A or Group B. Randomised means that a computer will allocate you randomly (as if by the roll of a dice) to receive: either A) support from a Community Navigator in addition to the support you already receive from mental health services; or B) no additional support, but you would carry on receiving your current support from mental health services. Neither your doctor, the study team, nor you will choose which treatment you receive, and sheer chance will decide who enters which group.

You will be asked to participate in a few research interviews: a) a research interview when you enter the study and before randomisation b) and another interview about 8 and 14 months later (each interview lasting around 1 hour). These interviews will involve completing questionnaires about loneliness, social support, activity and mood. The researcher who meets you for your follow-up interview will not know whether you were allocated to get a Community Navigator and will ask you to not to tell them if you were or not. You will also be asked to provide some brief information about your levels of depression, loneliness, and your use of community activities at 4 and 11 months after you join the study. This will take about ten minutes. You can provide this information yourself through a secure online form, or via a phone or video call with a researcher if you prefer. A researcher will also collect information from your NHS patient records including your diagnosis, care cluster, prescribed medication and use of community mental health and social care services. However, this will not need your active involvement. We will also seek your consent to collect any of this information from your GP records if it is not available from mental health services.

**Group A**

If you are allocated to Group A, you will be offered up to 10 meetings with a Community Navigator, over a six-month period. This will be in addition to the support you already receive from mental health services. Your Community Navigator will first of all support you to think about your existing social network, and the current and potential support it provides; your existing strengths and interests and any potential areas where you feel new activity, social connection or support would be beneficial. After your ten sessions, the Community Navigator will offer you one follow-up phone call and you will then be able to attend one more group meeting over the next two months.

Together with your Community Navigator, you can develop and use an action plan to increase how connected you are to your community. Examples of the types of support you could receive include practical help to access an activity you choose, local knowledge of leisure activities, culture or social support groups. The Community Navigator will also help you access and participate in social activities and to develop network connections. The Community Navigators are employed by the NHS. They have all received training, had background checks and will have supervision for this role.

The Community Navigators will also be running sessions open to everyone in Group A. There will be up to four meetings, alongside individual meetings with your Community Navigator, which will be a good chance to meet other people taking part in the programme. It will help you to understand more about this programme of support and give you a chance to share ideas about groups or activities that you enjoy.

If you are allocated to Group A, and receive support, a researcher will receive an anonymous account from your Community Navigator about what you are doing together in each of your meetings. No personal information will be included in this account, and you will not be identifiable from it.

If you are in Group A, you may also be asked to take part in a more in-depth research interview. In this interview, we are looking to hear what works well about the programme and what isn’t working so well, so that we can continue to improve the programme. It is your choice whether to take part in the interview and whether to provide feedback on your meetings. If you decide not to take part, it will not affect your relationship or the services that you receive from your Community Navigator or mental health services.

**Group B**

If you are allocated to Group B, you will continue with your usual support and will be given some written information about local resources and community activities.

**Taking Part in Community Navigator feasibility trial: What is involved?**

**What are the possible benefits of taking part?**

We hope that having support from a Community Navigator will reduce feelings of loneliness and improve quality of life and health. Reading through the written information about local resources and community activities may also be useful. People in both groups will be involved in a study which will help to find out more about what kind of support is helpful for people with long-term depression who are experiencing feelings of loneliness. People in both groups will be offered a £20 gift voucher for the initial interview and for the follow-up follow up research interviews 8 and 14 months later. (No payment will be offered for the brief information sought after 4 and 11 months.) If you were allocated to **Group A,** a study researcher may contact you to arrange an interview with you to explore in more detail how you experienced the support being offered.

**What are the possible disadvantages of taking part?**

It is possible that participants might not benefit from taking part in the study. You might not find receiving support from the Community Navigator, or the written information about local resources helpful, and this could be disappointing. Making a plan to engage more with people and activities in your community could be very useful but you may find it challenging. Making goals and feeling unable to meet them could be frustrating. However, the goals you choose to make and the activities you decide to participate in will be completely led by you. If you do not wish to continue meeting the Community Navigator, you are free to stop meeting at any point.

If you have enjoyed meeting the Community Navigator and found their support useful, you may be unhappy when your meetings end after 10 sessions. They will discuss with you where you could access ongoing support or information about local organisations and services. We hope this will help make the ending of this support easier.

If you do not receive support from the Community Navigator as you were randomly allocated to the control group of the study (Group B), this could be disappointing. You will receive written information about local groups and activities which you may like to attend, and you could discuss this with your care coordinator or another member of staff from the service you use.

**Will my participation be kept confidential?**

The mental health service that is supporting you and your GP will know you are taking part in the study. Community Navigators will make brief notes on your meetings which will be included in your NHS patient records.

Any information that is collected about you will be kept strictly confidential and anonymised. During this research we will collect your contact details, your responses to screening questions, and your response to interview questions and questionnaires. All the information we collect about you and the person you will be anonymised using a unique identification number so that it will not be possible to identify you from any of your information. Your data will be stored using this unique identification number and not your contact details (i.e., names or addresses) so that you cannot be identified from it. All data will be kept strictly confidential and will only be seen by members of the research team. The only exception to this would be if we received any information that indicates that you, or somebody else, are at risk of harm. In event of this we would contact the relevant authorities.

Two types of data will be collected from you and the person you care during this study; personal data so that we can contact you throughout your participation in the research and send you the study results if you have asked for this, and the research data which is the information we have collected from you during all the activities you have taken part in as part of the research. Personal data will be stored for no longer than we need to contact you, so until your participation in the study has ended or until we have written up the study results so that we can send them to you if you have requested this. Research data will be kept for up a minimum of 10 years so that the information can be verified at a later date if necessary.

**What will happen to my information?**

All of the information about you will be stored in one of two ways, electronic and paper copies. Some of the research data originally collected in paper format may be converted electronically so that we can store it on a secure computer network. All electronic data will be held on a secure database on a password-protected, encrypted computer and on University College London's password-protected, encrypted secure electronic network. Data in paper format (e.g., consent forms, completed questionnaires) will be stored securely in locked cabinets at the university where the researchers are based (University College London, University of Birmingham, or University of York) for up to three years and then sent away to be securely archived. All data will be securely destroyed after the retention period.

If any of the sessions that you have been involved in have been audio-recorded, once recordings have been transcribed (written up) they will be deleted.

Health care research is sometimes monitored to ensure that it is being conducted satisfactorily and that the interests of those taking part are protected. Such monitoring will be organised by the regulators responsible for healthcare research, the sponsor (Camden and Islington NHS Foundation Trust), the universities conducting the research, the funders (National Institute for Health Research) or the NHS trusts responsible for your mental health service. These monitors will need to look at information collected for this research which may include sections of your research notes. No personal information is ever collected.

**What will happen if I don’t want to carry on with the study?**

If you don't want to carry on with the study, you will be free to withdraw from it at any time, without having to give a reason. Withdrawing from the study will not affect the standard of care that you receive or your future medical care or legal rights. If you decided to withdraw from the study or if you lost capacity to make decisions and could not take part any more, then we would use any information collected in the study up to the point that you withdrew from the study, unless you tell us you don’t want us to do this.

**How will we use information about you?**

We will need to use information from you, from your medical records and from your GP for this research project. This information will include your:

- Initials
- NHS number
- Name
- Contact details

People will use this information to do the research or to check your records to make sure that the research is being done properly. People who do not need to know who you are will not be able to see your name or contact details. Your data will have a code number instead.  We will keep all information about you safe and secure. Once we have finished the study, we will keep some of the data so we can check the results. We will write our reports in a way that no-one can work out that you took part in the study.

**What are your choices about how your information is used?**

- You can stop being part of the study at any time, without giving a reason, but we will keep information about you that we already have.
- If you choose to stop taking part in the study, we would like to continue collecting information about your mental health service use from patient records. Data will be collected only for the period you were participating in the study. If you do not want this to happen, tell us and we will stop.
- We need to manage your records in specific ways for the research to be reliable. This means that we won’t be able to let you see or change the data we hold about you.

**Where can you find out more about how your information is used:**

You can find out more about how we use your information

- at [www.hra.nhs.uk/information-about-patients/](https://www.hra.nhs.uk/information-about-patients/)
- our leaflet available from <http://www.hra.nhs.uk/patientdataandresearch>
- by asking one of the research team
- by sending an email to the study researcher (millie.o’sullivan@candi.nhs.uk / gamze.evlat2@candi.nhs.uk)
- by ringing the study researcher on 07462 075906 / 07510 239172
- by sending an email to [information.request@candi.nhs.uk](mailto:information.request@candi.nhs.uk)

The data custodian for this study is Dr Bryn Lloyd-Evans.

**Local Data Protection Privacy Notice**

The controller for this study will be Camden & Islington NHS Foundation Trust and can be contacted at [sponsor.noclor@nhs.net](mailto:sponsor.noclor@nhs.net). The C&I Data Protection Officer provides oversight of activities involving the processing of personal data and can be contacted at  [information.request@candi.nhs.uk](mailto:information.request@candi.nhs.uk). University College London (UCL) will act as the Data Processor for this study. This ‘local’ privacy notice sets out the information that applies to this particular study. Further information on how UCL uses participant information can be found in our ‘general’ privacy notice. For participants in research studies, click here: https://www.ucl.ac.uk/legal-services/privacy/ucl-general-research-participant-privacy-notice

The information that is required to be provided to participants under data protection legislation (GDPR and DPA 2018) is provided across both the ‘local’ and ‘general’ privacy notices.

The categories of personal data used will be as follows:

- Name (on consent form only)
- Contact details
- Address to send report if wished
- Gender
- Age
- Ethnicity
- Marital status
- Accommodation

The lawful basis that will be used to process your personal data are: ‘Public task’ for personal data and’ Research purposes’ for special category data. Your personal data will be processed so long as it is required for the research project. We will endeavour to minimise the processing of personal data wherever possible. If you are concerned about how your personal data is being processed, or if you would like to contact us about your rights, please contact the C&I Data Protection Officer at  [information.request@candi.nhs.uk](mailto:information.request@candi.nhs.uk).

**What happens to the results of the research study?**

All of the information collected during the study will be made anonymous and written up in a report. The report will not contain any personal information from which you could be identified.

The study results are likely to be published in scientific journals and conferences. If you are interested in the study findings, we can send you a summary of these if you request this.

**Where can I get further information?**

If you require any further information or have any questions not answered by this information sheet, or if you have any comments or concerns, please do not hesitate to contact a member of the research team.

**Study researchers:**

[Name of McPin Peer Researcher]

Study researcher, The McPin Foundation

Email:

Tel:

[Name of Research Assistant]

Role, Affiliation

Email:

Tel:

The Trial Manager is responsible for the overall day-to-day management of the study and is based at University College London (UCL). The Research Assistants are based at the participating universities and will be in contact with potential participants to tell them about the study, take informed consent and conduct the study questionnaires with participants. If you decide to take part in the study, your main point of contact will be the Research Assistant that recruited you. The peer-researcher (researcher with lived experience) employed by the McPin Foundation will conduct qualitative interviews with participants who agree to this. An additional information sheet and consent form are provided for qualitative interview participants.

**What if I am unhappy with the research?**

If you have any concerns about the way, you have been treated during the course of the research, the researcher will be very happy to discuss this with you. You could also contact the Chief Investigator:

**Professor Brynmor Lloyd-Evans**

*Division of Psychiatry, University College London*

Email: [b.lloyd-evans@ucl.ac.uk](mailto:b.lloyd-evans@ucl.ac.uk)

Tel: [number]

If you wish to complain formally, or have any unresolved concerns about any aspect of the way you have been approached or treated during the course of this study, you can contact your local NHS Advice and Complaints Service:

**Advice and Complaints Service**

[Name of the service]

Address

Tel:

E-mail:

**Who is organising and funding the research?**

The research is sponsored by Camden and Islington NHS Foundation Trust. The research team are based atUCL, the Universities of Birmingham and York, and The McPin Foundation. The McPin Foundation is a not-for-profit organisation which supports service user involvement in mental health research. A peer-researcher employed by the McPin Foundation will conduct qualitative interviews with participants who agree to this.The study is funded by the NHS National Institute for Health Research, through its Health Technology Assessment programme.

**Who has reviewed the study?**

This study has been reviewed by the South Central – Oxford B Research Ethics Committee and was approved on 30^th^ March 2022 (REC reference 22/SC/0064).

**Thank you for reading this information sheet**
